# Supplementary material for: The role of mechano-regulated YAP/TAZ in erectile dysfunction
Source: Nat Commun. 2023 Jun 23;14:3758. doi: 10.1038/s41467-023-39009-z (PMC10290143; doi:10.1038/s41467-023-39009-z)
Supplement: Supplementary file 1 — Supplementary Information [file 41467_2023_39009_MOESM1_ESM.pdf]

Supplementary Information

The Role of Mechanoregulated YAP/TAZ in Erectile Dysfunction.

Mintao Ji[1]†, Dongsheng Chen[2]†, Yinyin Shu[1], Shuai Dong[1], Zhisen Zhang[1], Haimeng Zheng[1], Xiaoni Jin[1], Lijun Zheng[1], Yang Liu[3], Yifei Zheng[4,5], Wensheng Zhang[6], Shiyou Wang[2], Guangming Zhou[1], Bingyan Li[7], Baohua Ji[4], Yong Yang[3]*, Yongde Xu[8]*, Lei Chang[1]*

*Corresponding Email: Yong Yang: yangyong@ccucm.edu.cn; Yongde Xu: xyongd@yeah.net; Lei Chang: changlei@suda.edu.cn.

†These authors contributed equally to this work.

This PDF file includes:

Supplementary Figures 1 to 8

Supplementary Table 1

Supplementary Fig. 1. An erect penis mechanically stretches SMCs and activates YAP/TAZ.

a, Representative images (left) and quantification (right) of the ICP from erectile and flaccid penises of mice (n=7). **b**, Violin plots of representative markers for each cell type in single nucleus RNA sequencing (snRNAseq) data sets. **c**, Heatmap plots of gene modules specifically expressed in each cell type. **d**, Transcription factor motif enriched in promoter regions of gene sets differentially expressed between flaccid and erection conditions of SMCs. **e**, GSEA analyze of YAP/TAZ target genes between flaccid and erection conditions of cell clusters. Box plots indicate median (middle line), 25th, 75th percentile (box), minima, maxima and all points. Each point represents a mouse biologically independent sample and the experiments were repeated three independent times with similar results. The statistical analysis was calculated by two-sides unpaired Student's *t*-test, the confidence interval is 95%. Source data are provided as a Source Data file.

Supplementary Fig. 2. YAP/TAZ plays a vital role in rat ED models.

a, Immunofluorescence images of YAP/TAZ (red), αSMA (green) and DAPI (blue) and immunohistochemistry of TAZ from sham rats and the indicated time points of the BCNI-induced rat ED models. Quantification of TAZ immunohistochemistry (**b**) from sham rats (n=3) and 5 days (n=5), 14 days (n=3), 28 days (n=5) and 60 days (n=5) indicated time points of the BCNI-induced rat ED models. Expression of the YAP/TAZ target gene *Cyr61* (**c**) from sham rats (n=3) and 5 days (n=6), 14 days (n=4), 28 days (n=6) and 60 days (n=7) indicated time points of the BCNI-induced rat ED models. **d**, Immunofluorescence images of YAP/TAZ (red), αSMA (green) and

DAPI (blue) and immunohistochemistry of TAZ from the control and indicated time points of the IR-induced rat ED models. Quantification (**e**) of TAZ from control (n=5) and 1 month (n=3), 2 months (n=5), 3 months (n=5) and 5 months (n=7) indicated time points of the IR-induced rat ED models. Expression of the YAP/TAZ target gene *Cyr61* (**f**) from control (n=16) and 1 month (n=3), 2 months (n=5), 3 months (n=6) and 5 months (n=10) indicated time points of the IR-induced rat ED models. Data are presented as the mean ± sem. Each point represents a biologically independent rat sample and the experiments were repeated three independent times with similar results. The statistical analysis was calculated by two-sides unpaired Student's *t*-test, the confidence interval is 95%. Scale bars, 10 μm. Source data are provided as a Source Data file.

Supplementary Fig. 3. Mechanical stretching exerts a predominant effect on YAP/TAZ activity in comparison to PDE5i.

a, Quantification of YAP/TAZ (red) and DAPI (blue) in SMCs replated on a high or low mechanical ECM (n=16). Quantification (**b**) of YAP/TAZ (red) and DAPI (blue) in control (n=6) SMCs treated with the indicated mechanotransduction inhibitors, such as Dasa (dasatinib, n=10), Y27632 (n=5), and latrunculin A (Lat.A, n=8). Immunofluorescence images (**c**) of YAP/TAZ (red) and DAPI (blue), quantification (**d**) of YAP/TAZ localization in SMCs replated on the sparse (PDE5i untreated, n=11; PDE5i treated, n=9) or dense (PDE5i untreated, n=11; PDE5i treated, n=11) states and treated with/without PDE5i. Expression of the YAP/TAZ target gene *Ctgf* (**e**) and *Cyr61* (**f**) in SMCs replated on the sparse or dense states and treated with/without PDE5i (n=4). Immunofluorescence images (**g**) of YAP/TAZ (red) and DAPI (blue), quantification (**h**) of YAP/TAZ localization in control (n=5) SMCs treated with Ble (n=7) or Jas (n=13). Expression of the YAP/TAZ target gene *Ctgf* (**i**) and *Cyr61* (**j**) in SMCs treated with Ble or Jas (n=4). Immunofluorescence images (**k**) of YAP/TAZ (red) and DAPI (blue), quantification (**l**) of YAP/TAZ localization in control (n=5) SMCs treated with Lat.A (n=13) or Myo A (n=10). Expression of the YAP/TAZ target gene *Ctgf* (**m**) and *Cyr61* (**n**) in SMCs treated with Lat.A or Myo A (n=4). Box plots indicate median (middle line), 25th, 75th percentile (box), minima, maxima and all points. Dot plots indicate mean (middle line) and all points. Bar charts are presented as the mean ± sem. The statistical analysis was calculated by two-sides unpaired Student's *t*-test, the confidence interval is 95%. The point represents a mouse or detected cell area over 3 biologically independent experiments with similar results. Scale bars, 10 μm. Source data

are provided as a Source Data file.

Supplementary Fig. 4. ADM is transcriptionally regulated by the mechano-YAP/TAZ axis and independent of PDE5i.

Heatmap showing the changes in gene expression (**a**) and table of the most changed genes (**b**) in primary penile SMCs treated with siYAP/TAZ. **c**, qRT-PCR assessing the expression of *Yap*, *Taz*, *Amotl2*, *Ptx3*, *Adm, and P2x5* from RNA-seq. n = 2 biologically independent samples. **d**, qRT-PCR assessing the interaction sites between YAP and the ADM promotor. **e**, Schematic of the binding site of YAP and the ADM promotor. Bar charts are presented as the mean. Source data are provided as a Source Data file.

Supplementary Fig. 5. YAP/TAZ-ADM controls the penile SMCs' contraction.

a, Representative flow cytometry images of Ca^{2+} level in primary penile SMCs treated with Ad-Cre to knock out YAP/TAZ. **b**, Representative immunofluorescence of pMLC (green), phalloidin (red) and DAPI (blue) in primary penile SMCs treated with Ad-Cre to knock out YAP/TAZ. Representative immunofluorescence (**c**) and quantifications (**d**) of pMLC (green), phalloidin (red) and DAPI (blue) in control (n=42) primary penile SMCs treated with siYAP/TAZ (n=49), siADM (n=40), angiotensin II (as a positive control to increase contraction ability, n=39) and SNAP (as a positive control to decrease contraction ability, n=98), each point represents an area examined over 3 independent experiments. **e**, Representative contraction images (left) and quantifications of contraction ability (right) in control (n=11) primary penile SMCs treated with siADM (n=5) and siYAP/TAZ (n=3). **f**, Representative flow cytometry images of Ca^{2+} level in primary penile SMCs overexpressing ADM or knocking down YAP/TAZ by siRNA. **g**, Representative flow cytometry gating strategy of Ca^{2+} level in SMCs. **h**, Representative immunofluorescence of pMLC (green), phalloidin (red) and DAPI (blue) in primary penile SMCs overexpressing Flag-ADM or knocking down YAP/TAZ. **i**, Representative contraction images (upper) and quantifications of contraction ability (bottom) in control (n=8) primary penile SMCs overexpressing ADM (n=6) or knocking down YAP/TAZ (n=7) by siRNA. **j**, Contraction ability in control (n=5) primary penile SMCs overexpressing TAZ (n=5) or knocking down ADM (n=7). Representative images (**k**) and quantifications (**l**) of the ICP from Sham (n=8), BCNI (n=15), BCNI+PDE5i (Tadalafil, n=4), BCNI+PY60 (YAP/TAZ activator, n=8), BCNI+ADM mice (n=5), related to Fig. 3**v**. **m**, Expression of TAZ and ADM in the indicated groups. Box plots indicate median (middle line), 25th, 75th percentile (box), minima, maxima and all points. Violin and dot plots indicate mean

(middle line) and all points. Bar charts are presented as the mean ± sem. The statistical analysis was calculated by two-sides unpaired Student's *t*-test, the confidence interval is 95%. The point represents a mouse, sample or detected cell area over 3 biologically independent experiments with similar results. Scale bars, 10 μm. Source data are provided as a Source Data file.

Supplementary Fig. 6. VED promotes ED recovery by upregulating YAP/TAZ-ADM activity. ICP (left panel of **a**) and quantification (right panel of **a**) from the sham (n=8), BCNI (n=7) and BCNI+Vardenafil groups (n=5). Expression of the YAP/TAZ target gene *Cyr61* (**b**) in sham (n=5), BCNI (n=4), and BCNI combined VED (VED)-treated rats (n=6). Quantification of immunohistochemistry assessing the expression of TAZ (**c**) in sham (n=3), BCNI (n=5), and BCNI combined VED (VED)-treated rats (n=15). Representative immunofluorescence images (**e**) of YAP/TAZ (red), phalloidin (green) and DAPI (blue) and quantifications of YAP/TAZ localization (**d**) in sham (n=3), BCNI (n=3), and BCNI combined VED (VED)-treated rats (n=11). Box plots indicate median (middle line), 25th, 75th percentile (box), minima, maxima and all points. Dot plots indicate mean (middle line) and all points. Bar charts are presented as the mean ± sem. The statistical analysis was calculated by two-sides unpaired Student's *t*-test, the confidence interval is 95%. The point represents a mouse or rat sample over 3 biologically independent experiments with similar results. Scale bars, 10 μm. Source data are provided as a Source Data file.

Supplementary Fig. 7. SWT promotes ED recovery by upregulating YAP/TAZ-ADM activity. Expression of the YAP/TAZ target gene *Cyr61* (**a**) in sham (n=5), BCNI (n=4), and BCNI combined SWT (SWT) treated rats (n=7). Quantification of immunohistochemistry assessing the expression of TAZ (**b**) in sham (n=3), BCNI (n=3), and BCNI combined SWT (SWT) treated rats (n=15). Representative immunofluorescence images (**d**) of YAP/TAZ (red), phalloidin (green) and DAPI (blue) and quantifications of YAP/TAZ localization (**c**) in sham (n=3), BCNI (n=3), and BCNI combined SWT (SWT) treated rats (n=7). Representative image (**e**) and quantification (**f**) of MAP from the sham and *Yap* cKO mice (n=2, biologically independent samples). Representative immunofluorescence images (**g**) of Cre-GFP (green) in WT and $\mathrm{Cre}^{\mathrm{AAV}}$*Y/T* cKO mice. **h**, Western blots assessing the expression of TAZ and ADM in aorta or penis from WT, $\mathrm{Cre}^{\mathrm{AAV}}$*Y/T* cKO and $\mathrm{Cre}^{\mathrm{Ad}}$*Y/T* cKO mice. **i**, Western blots assessing the expression of YAP, TAZ and ADM from WT, $\mathrm{Cre}^{\mathrm{AAV}}$*Y/T* cKO and *Yap* cKO mice treated with/without VED. Dot plots indicate mean (middle line) and all points. Bar charts are presented as the mean ± sem. The statistical analysis was calculated by two-sides unpaired Student's *t*-test, the confidence interval is

95%. The point represents a mouse or rat sample over 3 biologically independent experiments with similar results. Scale bars, 10 µm. Source data are provided as a Source Data file.

Supplementary Fig. 8. The role of YAP/TAZ from pericyte or endothelial cells in erectile function and erectile restoration from the ED.

KEGG signaling pathways (left panel of **a**) and heatmap of YAP/TAZ target genes (right panel of **a**) between the erectile penis and flaccid penis of mice in pericytes. KEGG signaling pathways (left panel of **b**) and heatmap of YAP/TAZ target genes (right panel of **b**) between the erectile penis and flaccid penis of mice in endothelial cells. Representative immunofluorescence images (**c**) and quantifications (**d**) of immunofluorescence images of YAP/TAZ (red), PDGFRβ (green) and DAPI (blue) from sham rats (n=3) and 5 days (n=6), 14 days (n=3), 28 days (n=3) and 60 days (n=6) indicated time points of the BCNI-induced rat ED models. Representative immunofluorescence images (**f**) of YAP/TAZ (red), PDGFRβ (green) and DAPI (blue) and quantifications of YAP/TAZ localization (**e**) the sham (n=3), BCNI (n=6), and BCNI combined VED (VED) treated rats (n=6). Representative immunofluorescence images (left panel of **g**) of YAP/TAZ (red), PDGFRβ (green) and DAPI (blue) and quantifications of YAP/TAZ localization (right panel of **g**) in the sham (n=4), BCNI (n=3), and BCNI combined SWT (SWT) treated rats (n=5). Representative immunofluorescence images (**h**) of YAP/TAZ (red), CD31 (green) and DAPI (blue) in the sham, BCNI, BCNI+PDE5i, BCNI+PY60, BCNI+ADM mice. Dot plots indicate mean (middle line) and all points. The statistical analysis was calculated by two-sides unpaired Student's *t*-test, the confidence interval is 95%. The point represents a mouse or rat sample over 3 biologically independent experiments with similar results. Scale bars, 10 µm. Source data are provided as a Source Data file.

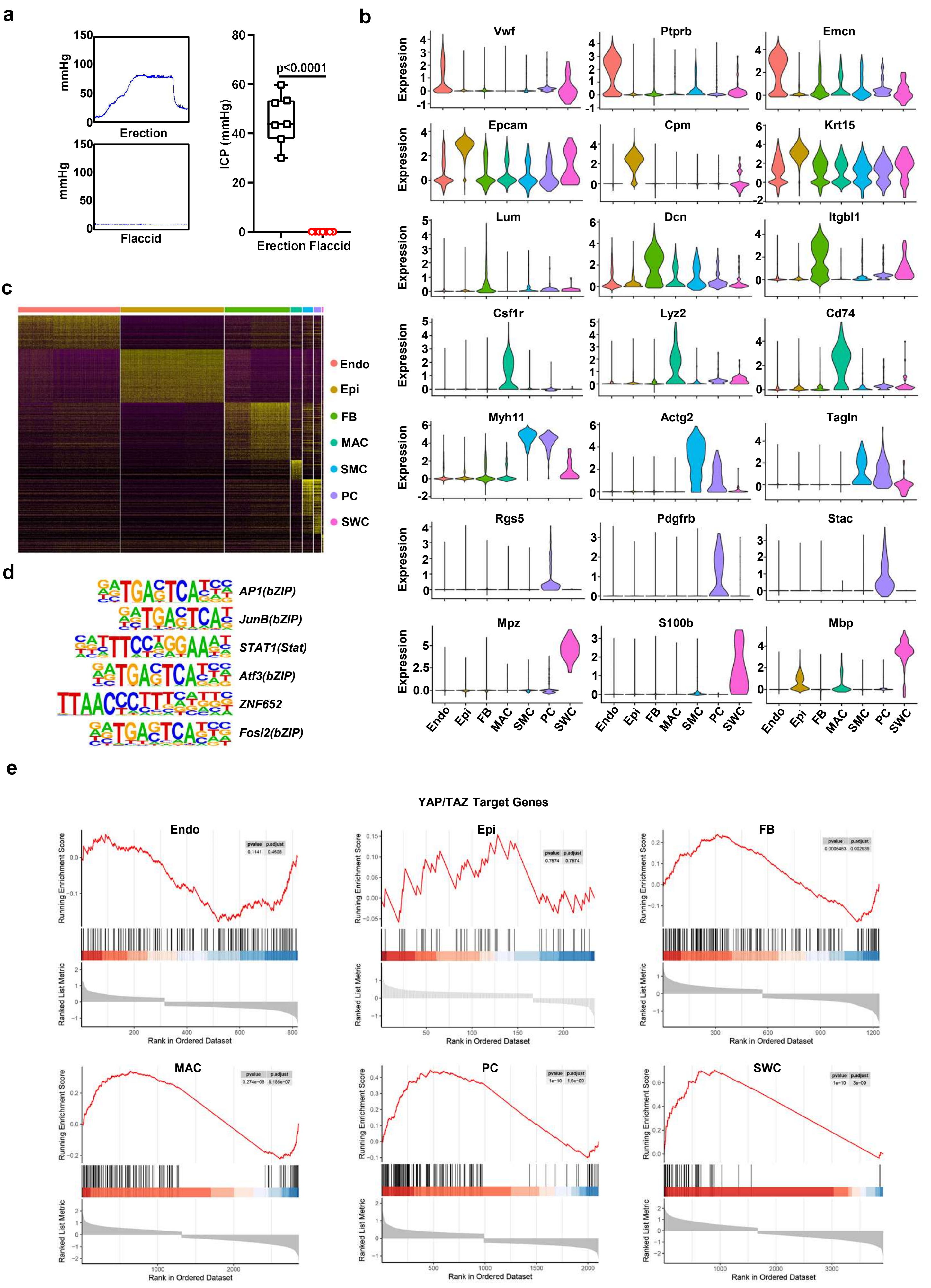

Supplementary Fig. 1. An erect penis mechanically stretches SMCs and activates YAP/TAZ.

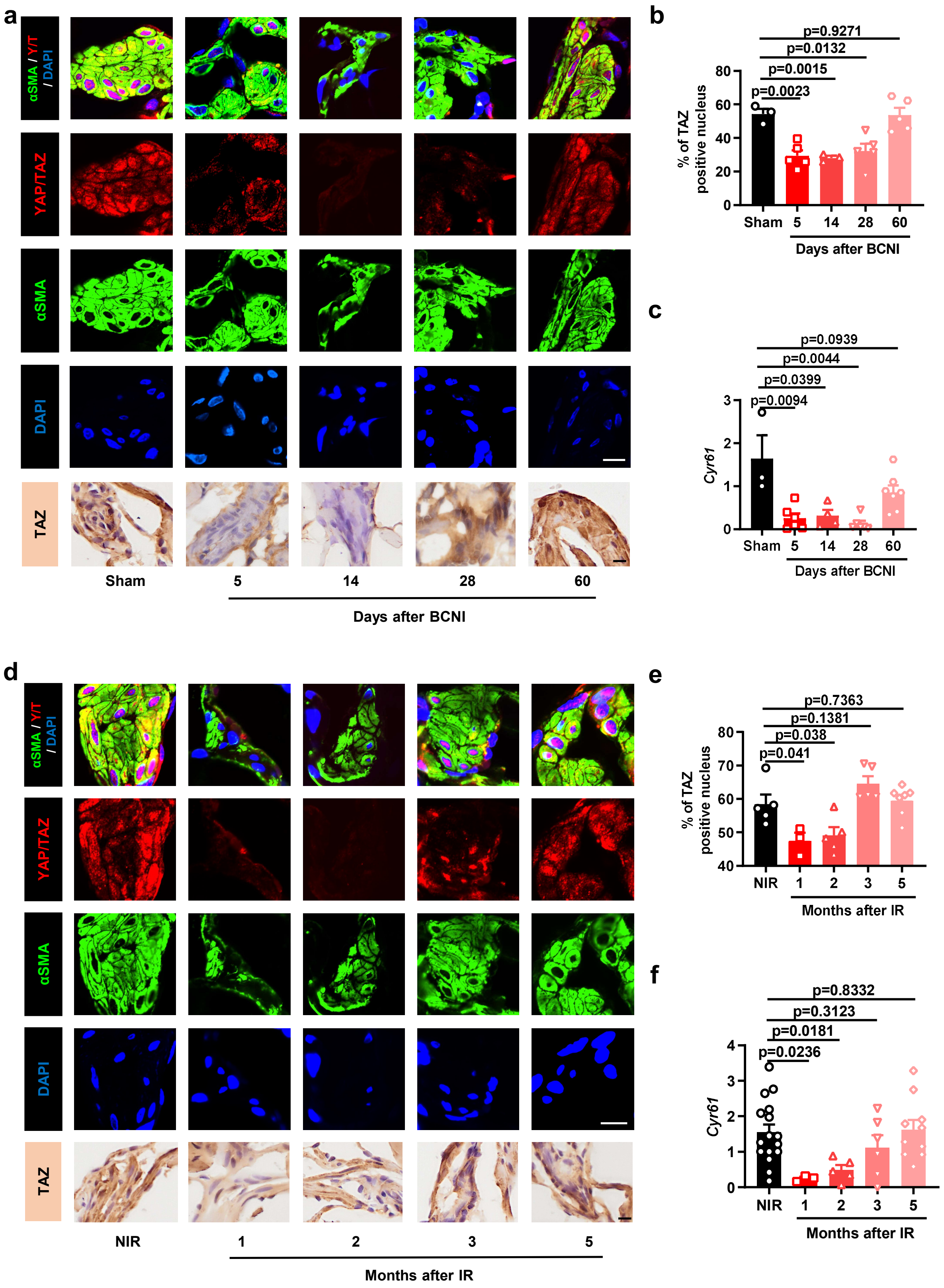

Supplementary Fig. 2. YAP/TAZ plays a vital role in rat ED models.

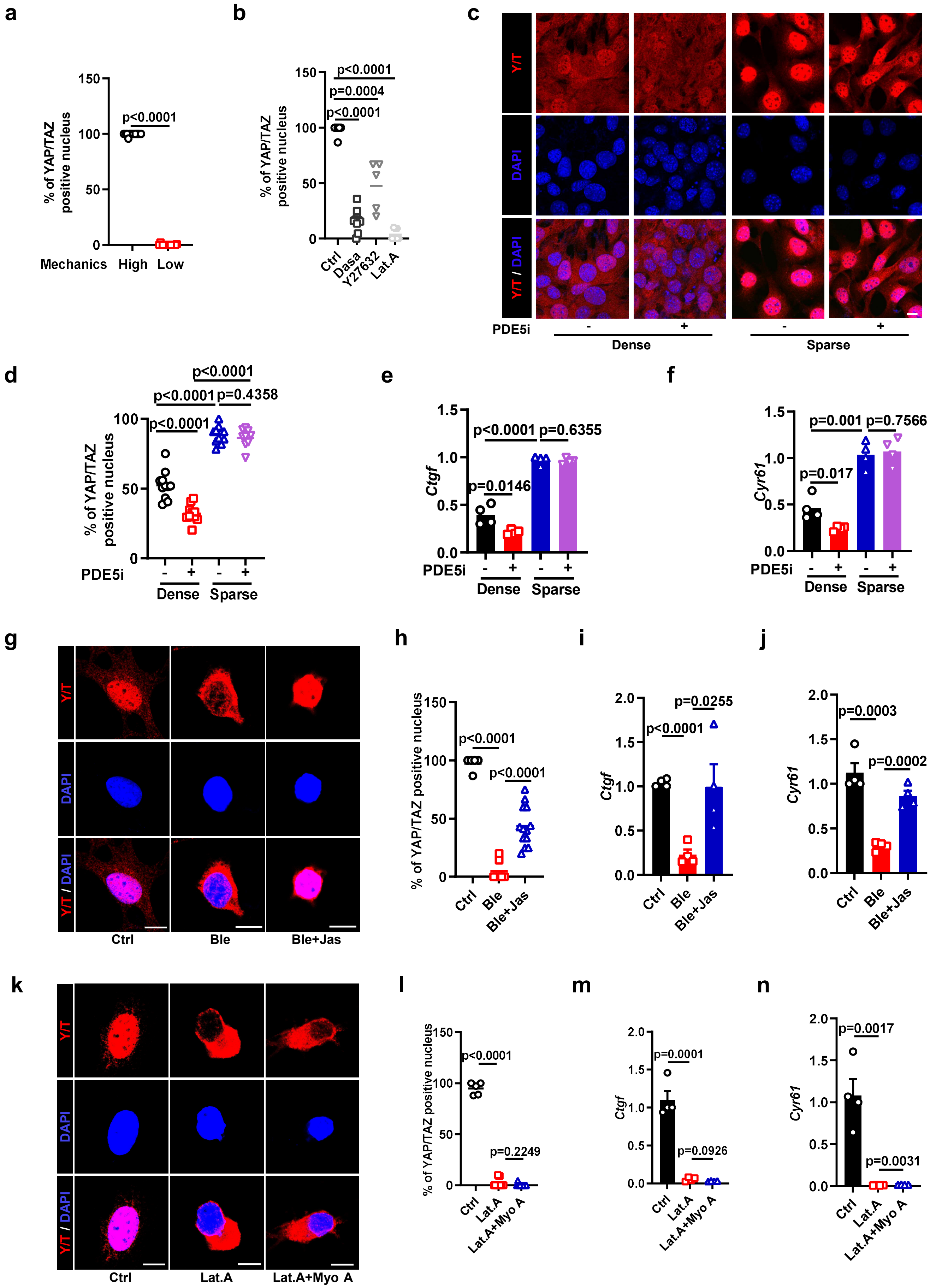

Supplementary Fig. 3. Mechanical stretching exerts a predominant effect on YAP/TAZ activity in comparison to PDE5i.

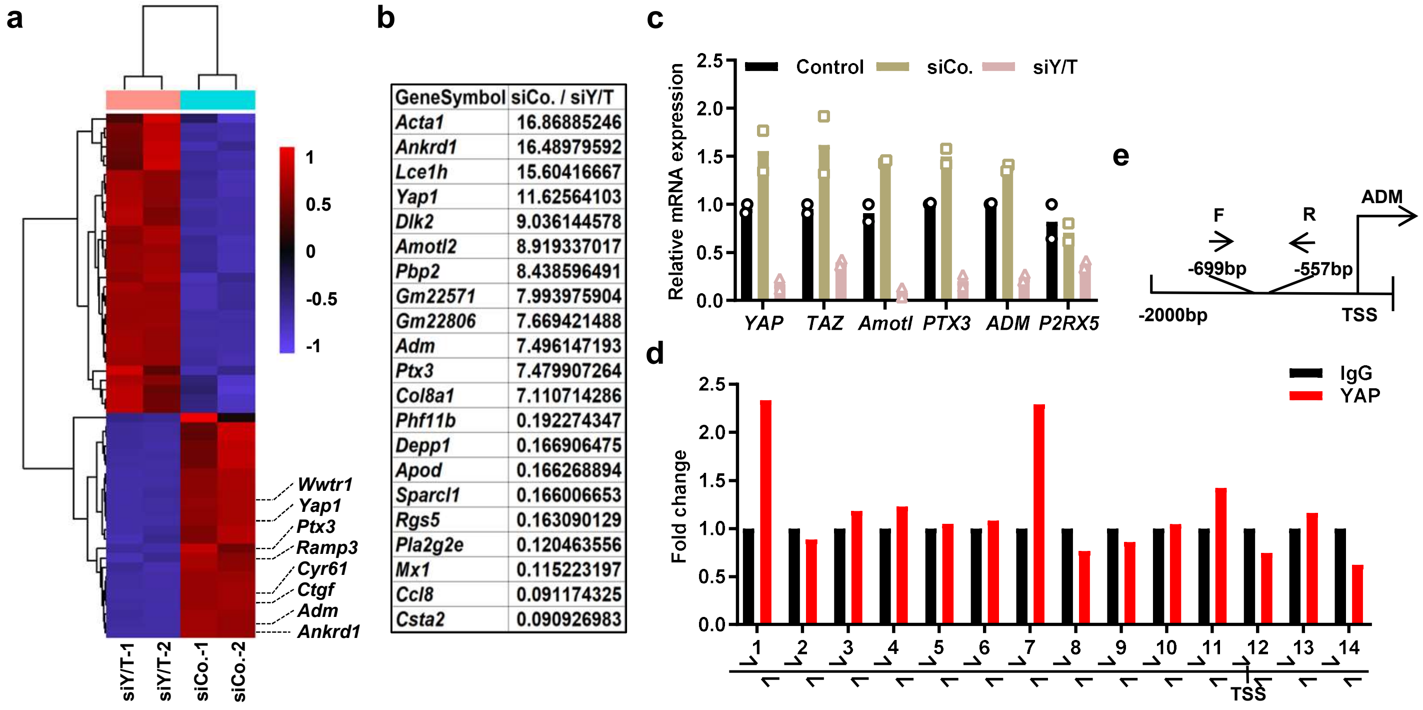

GeneSymbol	siCo. / siY/T
Acta1	16.86885246
Ankrd1	16.48979592
Lce1h	15.60416667
Yap1	11.62564103
Dlk2	9.036144578
Amotl2	8.919337017
Pbp2	8.438596491
Gm22571	7.993975904
Gm22806	7.669421488
Adm	7.496147193
Ptx3	7.479907264
Col8a1	7.110714286
Phf11b	0.192274347
Depp1	0.166906475
Apod	0.166268894
Sparcl1	0.166006653
Rgs5	0.163090129
Pla2g2e	0.120463556
Mx1	0.115223197
Ccl8	0.091174325
Csta2	0.090926983

Supplementary Fig. 4. ADM is transcriptionally regulated by the mechano-YAP/TAZ axis and independent of PDE5i.

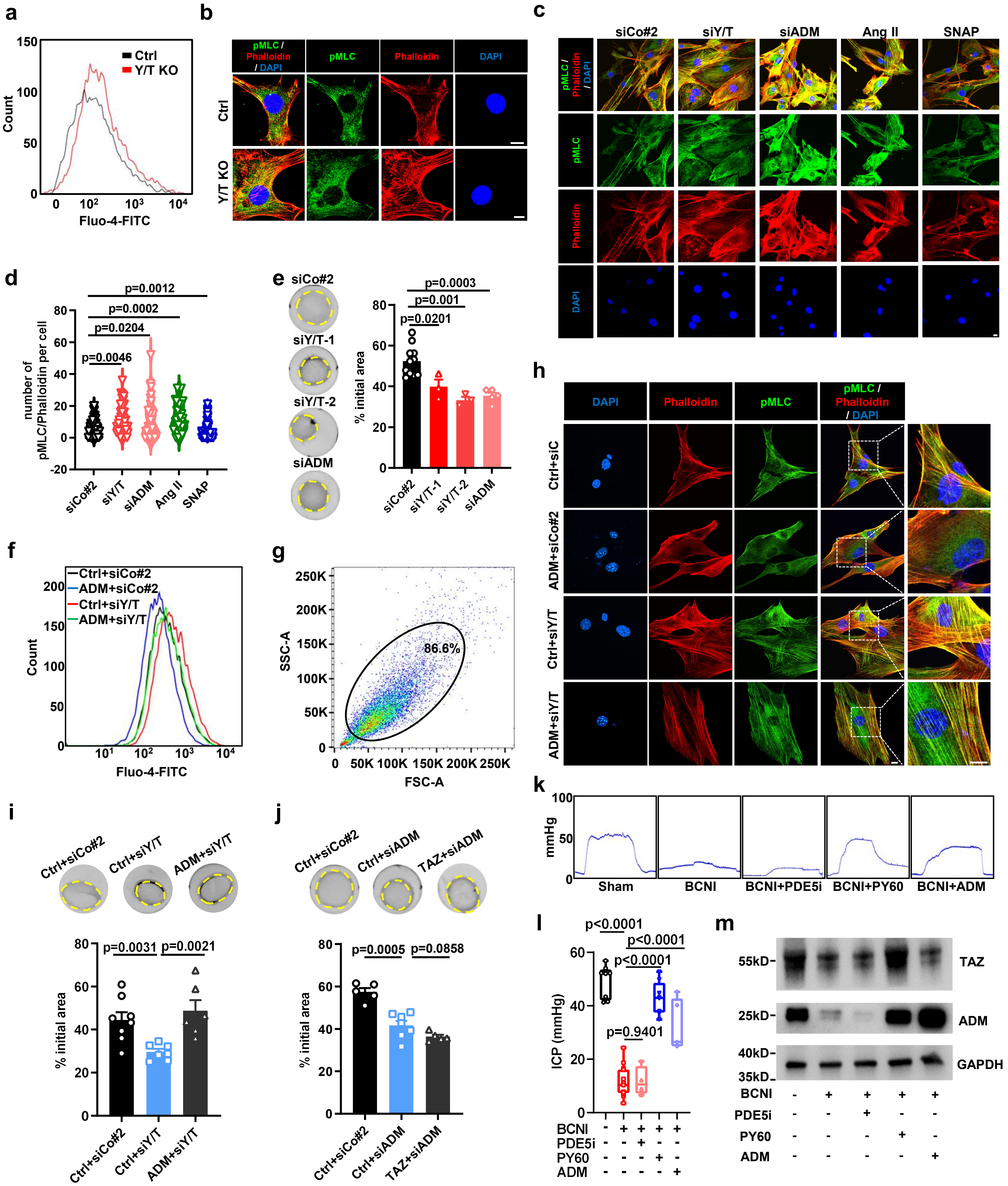

Supplementary Fig. 5. YAP/TAZ-ADM controls the penile SMCs' contraction

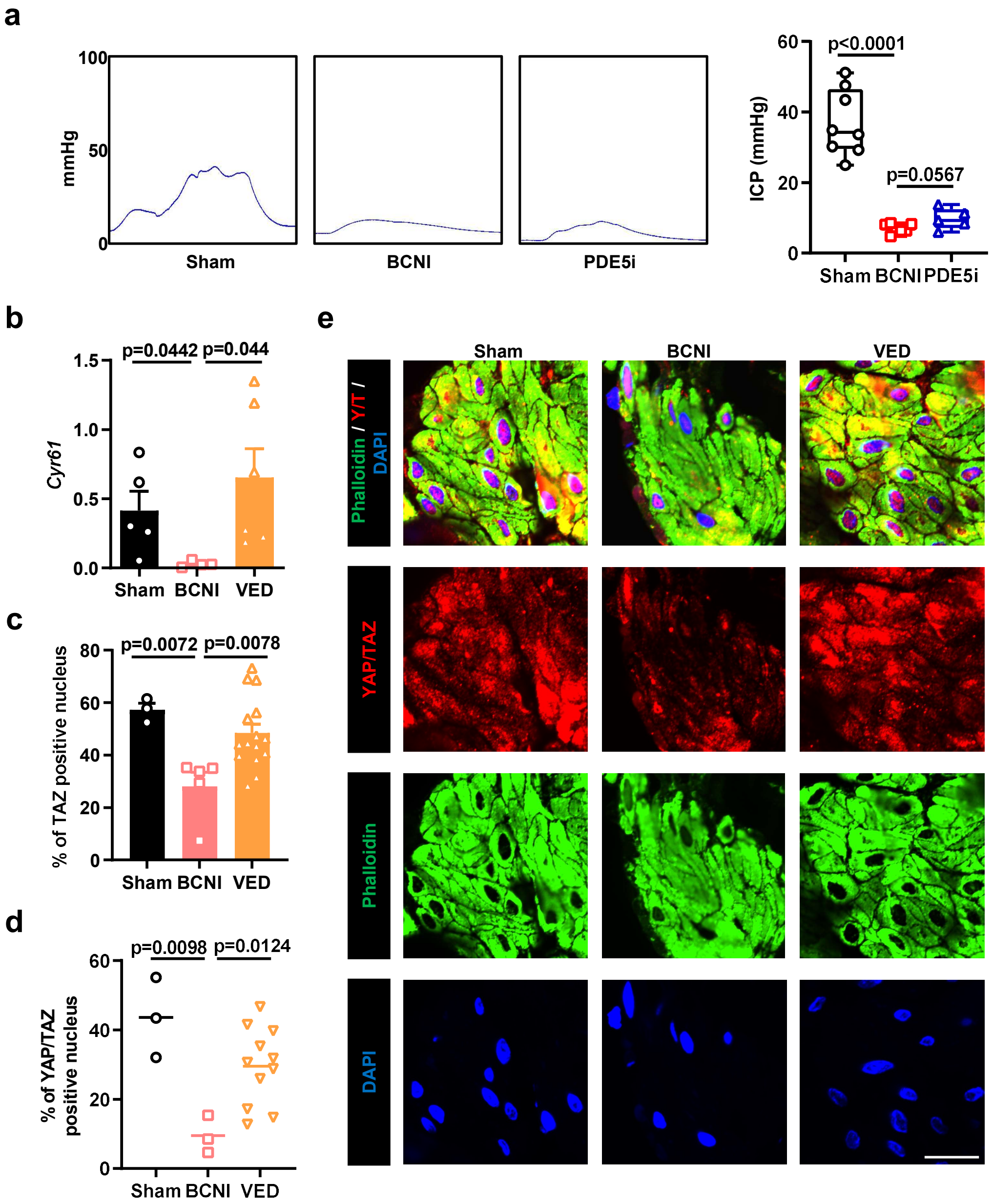

Supplementary Fig. 6. VED promotes ED recovery by upregulating YAP/TAZ-ADM activity.

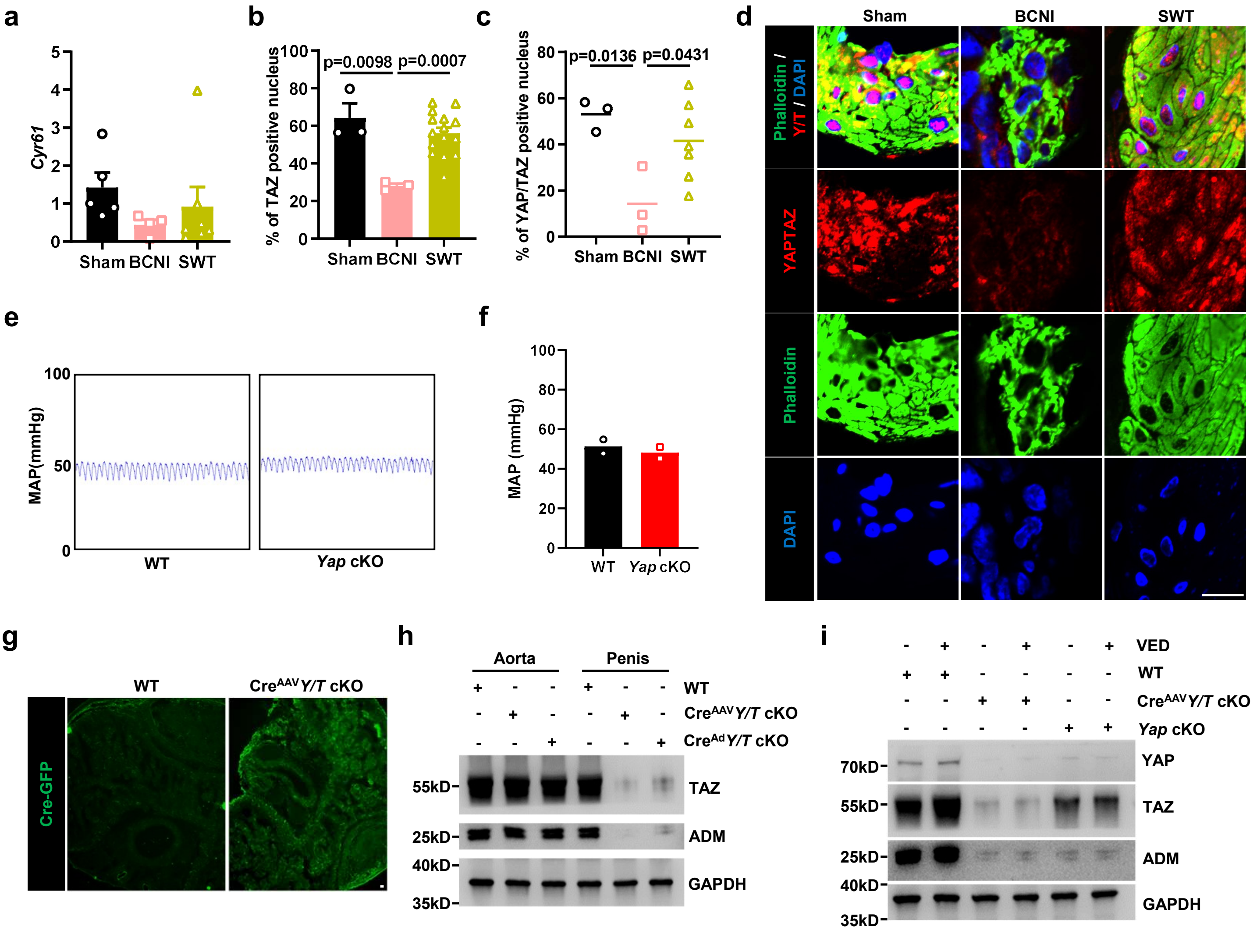

Supplementary Fig. 7. SWT promotes ED recovery by upregulating YAP/TAZ-ADM activity.

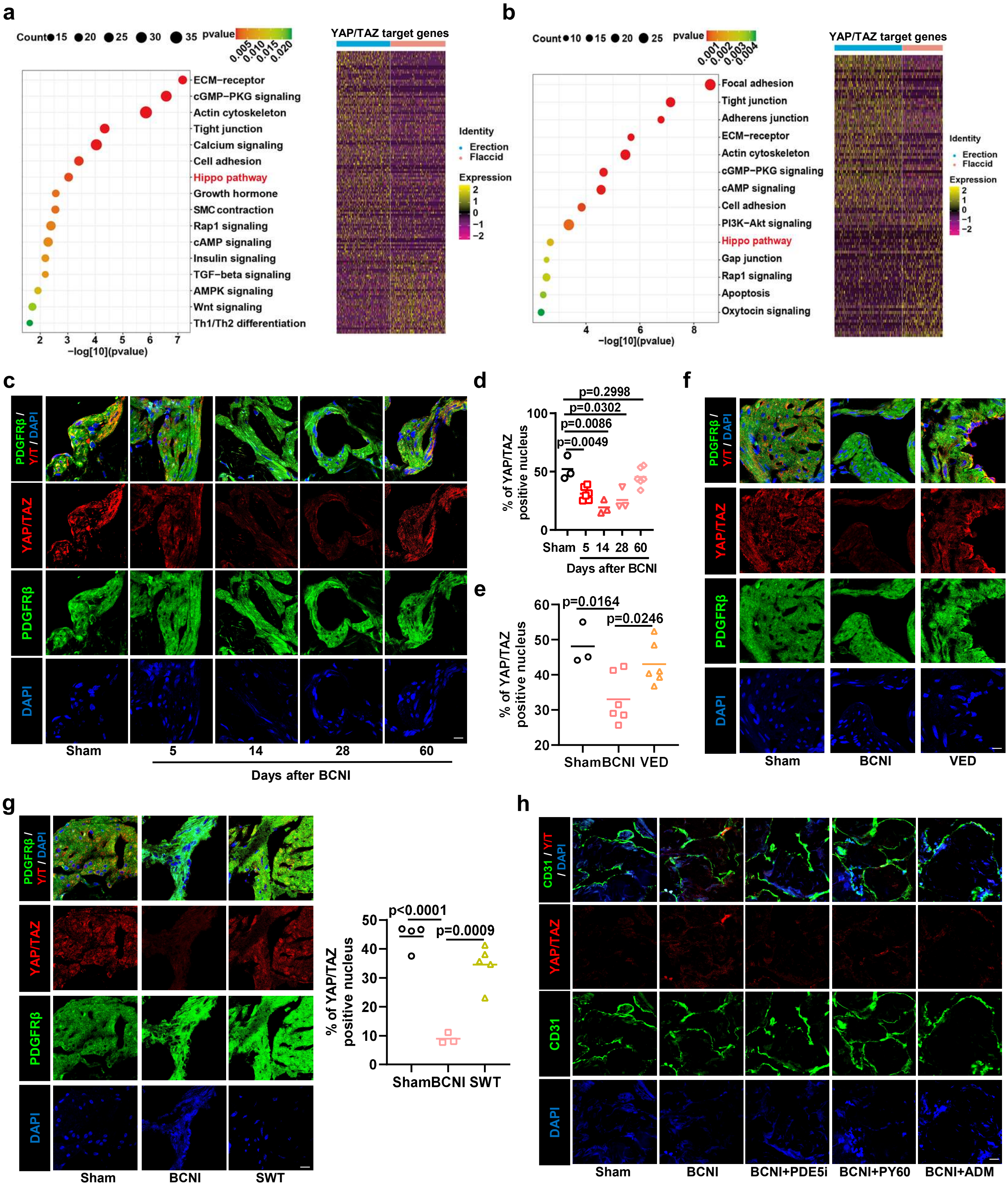

Supplementary Fig. 8. The role of YAP/TAZ from pericyte or endothelial cells in erectile function and erectile restoration from the ED.

**Supplemental Table1. PCR oligo sequences.**		
**Gene**	**Forward primer**	**Reverse primer**
Rat		
rCtgf	TCTTCGGTGGGTCCGTGTA	CGGTCCTTGGGCTCATCAC
rCyr61	AGAGGCTTCCTGTCTTTGG	CAGTATTTGGGCCGGTAT
rGapdh	TATCGGACGCCTGGTTAC	CTGTGCCGTTGAACTTGC
rAdm	TCGCCCTGATGTTATTGG	CTCTGAGTGCTGGCTTGTG
Mouse		
mTaz	ATGAATCCGTCCTCGGTGC	GAGTTGAAGAGGGCTTCGAG
mYap	AAGGAGAGACTGCGGTTGAA	CCTGAGACATCCCAGGAGAA
mGapdh	ATCCTGCACCACCAACTGCT	GGGCCATCCACAGTCTTCTG
mCtgf	CTGCCTACCGACTGGAAGAC	CATTGGTAACTCGGGTGGAG
mCyr61	GCTCAGTCAGAAGGCAGACC	GTTCTTGGGGACACAGAGGA
mAnkrd1	CTGTGAGGCTGAACCGCTAT	TCTCCTTGAGGCTGTCGAAT
mPtx3	GTGGTGGGTGGAAAGGAG	CCCGGATGTGACAGGATT
mAdm	TTGGGTTCACTCGCTTTC	ATGCCGTCCTTGTCTTTG
mAmotl2	GGAAAGAAACGGTGCTCG	GGTCGGTATCTTGGGTCAGT
mP2rx5	ACTGGTCGCTGTCTACGG	AGCACATTGGCTTTGGAG
mAdm-chip-1	GGGATAATAGTATCACAAGGA	TTCGTAATAAGGGCAGAA
mAdm-chip-2	TTAGAGGCAGATAAGGTAGG	TCCCGAAGCTAAAGACAT
mAdm-chip-3	TACCCTTTCGGCACTTCA	CTGCCTTCATTCATCCATCA
mAdm-chip-4	CCACCTGATGAGAATGGT	TAAAGTGAAGTGCCGAAA
mAdm-chip-5	AGTTCTGCCCTTATTACGA	CGGCTTAGCACCTGACTG
mAdm-chip-6	TGTGCTCCCAGTCAGTCA	GGAAATACAGGAATAGCCAAA
mAdm-chip-7	GTCGCCAGGAAGAGGACA	CCCTTCTAGGGCCAAACT
mAdm-chip-8	TTGGCCCTAGAAGGGAAGA	GCACAGCGGCTCGGTTTT
mAdm-chip-9	GTACCCTGCGGGCACAGAT	TGCAAGAACCGGGAGACG
mAdm-chip-10	GAGGACTCCAGGGCAAAT	CGATGAGAAGCCGAGAAA
mAdm-chip-11	AGTCCTGCTCCAGTCTAATC	CCAGCGTGCTTGCTTCTA
mAdm-chip-12	GTTTCTCGGCTTCTCATCG	GTCCAGCTCTGCAAGTTTT
mAdm-chip-13	CCTCCAGCATGTTAGGGA	GAGCTAGACCTGGGCAAT
mAdm-chip-14	TCAGCACTCTGGGTTGTAC	ATTAGACTGGAGCAGGACTT
mRps18-chip	AGTTCCAGCACATTTTGCGAG	TCATCCTCCGTGAGTTCTCCA

**Supplemental Table2. siRNA sequences.**	
siRNA	Interfering sequence (target)
siCo.	AllStars Negative Control siRNA
mouse	
	CAGCCGAATCTCGCAATGA (TAZ#1)
siY/T#1	CGGTTGAAACAACAGGAAT (YAP#1)
	GAAGCGCTGAGTTCCGAAA (YAP#2)
	CCATGAGCACAGATATGAG (TAZ#2)
siY/T#2	ACTTGGAGGCGCTCTTCAA (YAP#3)
	TGAGAACAATGACAACCAA (YAP#4)
