# Supplementary material for: The role of mechano-regulated YAP/TAZ in erectile dysfunction
Source: Nat Commun. 2023 Jun 23;14:3758. doi: 10.1038/s41467-023-39009-z (PMC10290143; doi:10.1038/s41467-023-39009-z)
Supplement: Supplementary file 3 — Reporting Summary [file 41467_2023_39009_MOESM3_ESM.pdf]

nature portfolio

Corresponding author(s): Lei Chang, Yongde Xu, Yong Yang

Last updated by author(s): Apr 12, 2023

Reporting Summary

Nature Portfolio wishes to improve the reproducibility of the work that we publish. This form provides structure for consistency and transparency in reporting. For further information on Nature Portfolio policies, see our Editorial Policies and the Editorial Policy Checklist.

Statistics

For all statistical analyses, confirm that the following items are present in the figure legend, table legend, main text, or Methods section.

n/a	Confirmed	
☐	☒	The exact sample size (*n*) for each experimental group/condition, given as a discrete number and unit of measurement
☐	☒	A statement on whether measurements were taken from distinct samples or whether the same sample was measured repeatedly
☐	☒	The statistical test(s) used AND whether they are one- or two-sided<br>*Only common tests should be described solely by name; describe more complex techniques in the Methods section.*
☐	☒	A description of all covariates tested
☐	☒	A description of any assumptions or corrections, such as tests of normality and adjustment for multiple comparisons
☐	☒	A full description of the statistical parameters including central tendency (e.g. means) or other basic estimates (e.g. regression coefficient) AND variation (e.g. standard deviation) or associated estimates of uncertainty (e.g. confidence intervals)
☐	☒	For null hypothesis testing, the test statistic (e.g. *F*, *t*, *r*) with confidence intervals, effect sizes, degrees of freedom and *P* value noted<br>*Give P values as exact values whenever suitable.*
☐	☒	For Bayesian analysis, information on the choice of priors and Markov chain Monte Carlo settings
☐	☒	For hierarchical and complex designs, identification of the appropriate level for tests and full reporting of outcomes
☒	☐	Estimates of effect sizes (e.g. Cohen's *d*, Pearson's *r*), indicating how they were calculated

Our web collection on statistics for biologists contains articles on many of the points above.

Software and code

Policy information about availability of computer code

Data collection	Flow cytometry acquisitions were performed using BD FACSverse. Code:Z6511550318.<br>Microscopy acquisition were performed using OLYMPUS FV1200.<br>ICP were performed using Acqknowledge software (Biopac System Inc., Goleta, CA, USA).
Data analysis	Image J software 1.51K was used to analyze most of the data including western blot, IHC, IF and contraction assay. Statistical tests were performed using excel (2019 MSO (16.0.14326.20450)). Flow cytometry data were analyzed using FlowJo V10.8.1 (M11c3c353YH92SCS). ICP were performed using Acqknowledge software (Biopac System Inc., Goleta, CA, USA). sn-RNA seq data analyzed using Seurat package 4.3.0.

For manuscripts utilizing custom algorithms or software that are central to the research but not yet described in published literature, software must be made available to editors and reviewers. We strongly encourage code deposition in a community repository (e.g. GitHub). See the Nature Portfolio guidelines for submitting code & software for further information.

Data

Policy information about availability of data

All manuscripts must include a data availability statement. This statement should provide the following information, where applicable:

- Accession codes, unique identifiers, or web links for publicly available datasets
- A description of any restrictions on data availability
- For clinical datasets or third party data, please ensure that the statement adheres to our policy

Single nucleus RNA sequencing data from rats and RNA sequencing data from mice can be accessed from the NCBI Gene Expression Omnibus database GSE208293 (https://www.ncbi.nlm.nih.gov/geo/query/acc.cgi?acc=GSE208293). The mouse genome (mm10) was downloaded from the FTP of the Ensembl database (https://asia.ensembl.org/Mus_musculus/Info/Index). All other relevant data supporting the key findings of this study are available within the article and its Supplementary Information files. Source data are provided with this paper.

Human research participants

Policy information about studies involving human research participants and Sex and Gender in Research.

Reporting on sex and gender	The patients are all male because our study field is based on erectile function
Population characteristics	The age of patients range from 18-80, and all the patients were diagnosed as erectile dysfunction. All the patients were treated by VED.
Recruitment	Our patients data were collected from the Department of Urology, Affiliated Hospital of Changchun University of Traditional Chinese Medicine, during 2020.1-2021.12. We collected these patients information under the below conditions.<br>Patients Selection Reason<br>1. Subject inclusion criteria:<br>a. male aged 18-80;<br>b. General health;<br>c. Consistent with the diagnosis of penile erectile dysfunction (ED);<br>d. Have a history of ED for at least 6 months and did not use PDE5i within 4 weeks before VED treatment;<br>e. The international erectile function score (IIEF-EF) is between 11 and 25;<br>f. Stable heterosexual relationship at least 3 months before treatment;<br>g. Patients with penile cancer who are to undergo partial penile resection and have normal penile erection.<br>h. Patients with ED who have failed to receive conservative treatment and plan to receive penile prosthesis implantation.<br>i. The subjects voluntarily agreed to participate and signed the informed consent.<br>2. Subject exclusion criteria:<br>a. ED caused by psychopathological factors;<br>b. Any unstable medicine, psychosis, spinal cord injury, penis anatomy abnormality;<br>c. Arterial obstruction is located in common penile artery and cavernous artery;<br>d. clinically significant chronic hematological diseases;<br>e. Venous leakage has been confirmed;<br>f. Poor blood pressure control (systolic blood pressure ≥ 160mmhg and diastolic blood pressure ≥ 100mmhg after treatment);<br>g. Coagulation dysfunction and use of anticoagulants (such as Coumadin);<br>h. Have participated in any other medical device or drug clinical research within three months;
Ethics oversight	The study was approved by the Affiliated Hospital of Changchun University of traditional Chinese Medicine ethics committee and complied with all relevant ethical regulations (License No. CCZYFYLL2019sz.063)

Note that full information on the approval of the study protocol must also be provided in the manuscript.

Field-specific reporting

Please select the one below that is the best fit for your research. If you are not sure, read the appropriate sections before making your selection.

☒ Life sciences ☐ Behavioural & social sciences ☐ Ecological, evolutionary & environmental sciences

For a reference copy of the document with all sections, see nature.com/documents/nr-reporting-summary-flat.pdf

Life sciences study design

All studies must disclose on these points even when the disclosure is negative.

Sample size	No statistical method was used to predetermine sample size. For cell related assay, at least 100 cells were analyzed to determine YAP/TAZ levels, and the experiments had three biological independent times with similar results. For in vivo study, each experiments exhibited at least 3 biological replicates of mice or rats to confirm our results.

Data exclusions	We did not exclude any data.
Replication	All experiments were repeated three independent times with similar results.
Randomization	All experiments were randomized grouping.
Blinding	The experiments in our study are in a blind manner, such as ICP measure, VED treatments.

Reporting for specific materials, systems and methods

We require information from authors about some types of materials, experimental systems and methods used in many studies. Here, indicate whether each material, system or method listed is relevant to your study. If you are not sure if a list item applies to your research, read the appropriate section before selecting a response.

Materials & experimental systems

n/a	Involved in the study
☐	☒ Antibodies
☐	☒ Eukaryotic cell lines
☒	☐ Palaeontology and archaeology
☐	☒ Animals and other organisms
☒	☐ Clinical data
☒	☐ Dual use research of concern

Methods

n/a	Involved in the study
☒	☐ ChIP-seq
☐	☒ Flow cytometry
☒	☐ MRI-based neuroimaging

Antibodies

Antibodies used	The antibodies used for western blot were: anti-YAP/TAZ (Santa Cruz Biotechnology, sc-101199 (1:1000)), anti-YAP1 antibody (Proteintech, Cat#13584-1-AP (1:300)), anti-WWTR1 antibody (ATLAS, Cat#HPA007415 (1:300)) and anti-ADM antibody (Abcam, Cat#ab190819 (1:300)) and anti-GAPDH (Millipore, MAB374 (1:30000)). The secondary antibodies were from Beyotime Biotechnology (anti-mouse: Cat# A0216 (1:500), anti-rabbit: Cat# A0208 (1:500)).<br>Primary IF antibodies against YAP/TAZ (Santa Cruz Biotechnology Cat# sc-101199 (1:300)), phospho-MLC (Cell Signaling Technology Cat# 3671 (1:300)), αSMA (Abcam Cat# ab124964 (1:300)), PDGFRβ (Cell Signaling Technology Cat# 3169 (1:300)), CD31 (Cell Signaling Technology Cat# 77699 (1:300)), and phalloidin (PHDH1) were purchased from cytoskeleton. Secondary antibodies (1:300) were obtained from Beyotime Biotechnology (anti-mouse: Cat# A0460 and A0428, anti-rabbit: Cat# A0423 and A0453). Samples were counterstained with Prolong-DAPI (Molecular Probes, Life Technologies) to label cell nuclei.<br>The antibodies for IHC were as follows: anti-YAP1 antibody (Proteintech, Cat# 13584-1-AP (1:300)), anti-WWTR1 antibody (ATLAS, Cat# HPA007415 (1:300)) and anti-ADM antibody (Abcam, Cat# ab190819 (1:300)).
Validation	anti-YAP/TAZ (Santa Cruz Biotechnology, sc-101199 (1:1000)) Validated for detecting cytoplasm and nucleus of SMCs<br>anti-YAP1 antibody (Proteintech, Cat#13584-1-AP (1:300)) Validated for detecting nucleus of corpus cavernosum<br>anti-WWTR1 antibody (ATLAS, Cat#HPA007415 (1:300)) Validated for detecting nucleus of corpus cavernosum<br>anti-ADM antibody (Abcam, Cat#ab190819 (1:300)) Validated for detecting cytoplasm of SMCs<br>anti-GAPDH (Millipore, MAB374 (1:30000)) used as the loading control<br>anti-phospho-MLC (Cell Signaling Technology Cat# 3671 (1:300)) Validated for detecting cytoskeleton of SMCs<br>anti-αSMA (Abcam Cat# ab124964 (1:300)) Validated for detecting cytoplasm of corpus cavernosum<br>anti-PDGFRβ (Cell Signaling Technology Cat# 3169 (1:300)) Validated for detecting cytoplasm of corpus cavernosum<br>anti-CD31 (Cell Signaling Technology Cat# 77699 (1:300)) Validated for detecting cytoplasm of corpus cavernosum

Eukaryotic cell lines

Policy information about cell lines and Sex and Gender in Research

Cell line source(s)	MOVAS(BNCC338213), HEK293T(CRL-3216), Primary penile smooth muscle cells were isolated by our lab.
Authentication	Cells were authenticated using the institut curie genotype validation
Mycoplasma contamination	All cells were routinely tested for Mycoplasm contamination. All cells used in this paper were mycoplasm-free.
Commonly misidentified lines (See ICLAC register)	We did not use any mis-identified cell line.

Animals and other research organisms

Policy information about studies involving animals; ARRIVE guidelines recommended for reporting animal research, and Sex and Gender in Research

Laboratory animals	C57 BL/6 mice and SD rats were wide type background<br>B6. FVB-Tg(Myh11-cre/ERT2)1Soff/J (Jackson lab: 019079).<br>STOCK Wwtr1tm1Hmc Yap1tm1Hmc/WranJ (Jackson lab: 030532).<br>Approximately 250 g male SD rats and 6- to 8-week-old male mice were used in this study.
Wild animals	This study did not involved wild animals.
Reporting on sex	The mice used in this study are male.
Field-collected samples	This study did not involved samples collected in the field.
Ethics oversight	All studies involving mice and rats were studied and operated followed by the rules of the Soochow University Institutional Animal Care and Use Committee.

Note that full information on the approval of the study protocol must also be provided in the manuscript.

Flow Cytometry

Plots

Confirm that:

- [x] The axis labels state the marker and fluorochrome used (e.g. CD4-FITC).
- [x] The axis scales are clearly visible. Include numbers along axes only for bottom left plot of group (a 'group' is an analysis of identical markers).
- [x] All plots are contour plots with outliers or pseudocolor plots.
- [x] A numerical value for number of cells or percentage (with statistics) is provided.

Methodology

Sample preparation	Smooth muscle cells were removed from the culture medium, washed twice with PBS, then incubated with 1μM Fluo-4 Am (S1060, Beyotime) in 37? incubator for 30-60 minutes. Then the cells were washed once with PBS, digested with trypsin and collected, finally resuspended with 500ul PBS, and tested by flow cytometry (FACSVerse, BD).
Instrument	BD FACSverse. Code:Z6511550318.
Software	BD FACS Software
Cell population abundance	Post-sort analysis was performed to determine the purity of the sorted populations.
Gating strategy	Experimental samples were recorded by the mean/ medium fluorescence intensity in different groups.

- [x] Tick this box to confirm that a figure exemplifying the gating strategy is provided in the Supplementary Information.
